# Regulation of cellular senescence by innate immunity

**DOI:** 10.52601/bpr.2023.230032

**Published:** 2023-12-31

**Authors:** Jinxiu Hou, Yi Zheng, Chengjiang Gao

**Affiliations:** 1 Key Laboratory of Infection and Immunity, Shandong Province & Key Laboratory for Experimental Teratology, Ministry of Education, Shandong University, Jinan 250012, China; 2 Department of Immunology, the School of Basic Medical Sciences, Shandong University, Jinan 250012, China

**Keywords:** IFN-I, NF-κB, cGAS-STING, TLRs, NLRP3 inflammasome, Cell senescence

## Abstract

During the COVID-19 pandemic, the interplay between the processes of immunity and senescence is drawing more and more intensive attention. SARS-CoV-2 infection induces senescence in lung cells, failure to clear infected cells and increased presence of inflammatory factors could lead to a cytokine storm and acute respiratory disease syndrome (ARDS), which together with aging and age-associated disease lead to 70% of COVID-19-related deaths. Studies on how senescence initiates upon viral infection and how to restrict excessive accumulation of senescent cells to avoid harmful inflammation are crucially important. Senescence can induce innate immune signaling, and innate immunity can engage cell senescence. Here, we mainly review the innate immune pathways, such as cGAS-STING, TLRs, NF-κB, and NLRP3 inflammasome, participating in the senescence process. In these pathways, IFN-I and inflammatory factors play key roles. At the end of the review, we propose the strategies by which we can improve the immune function and reduce inflammation based on these findings.

## INTRODUCTION

Pathogen-associated molecular patterns (PAMPs) can be recognized by the pattern recognition receptors (PRRs) of the cell (Ablasser and Hur 2020; Aleynick *et al.* 2019; Lukhele *et al.* 2019), by which the innate immune system represents the first defense line of the organism. Undoubtedly, foreign or misplaced nucleic acids are a critically important signal received by the innate immune system (Akira *et al.* 2006). Once PAMPs are sensed by PRRs, an efficient signal transduction system is elicited, resulting in the induction of type I interferons (IFN-I) and proinflammatory cytokines (Zheng and Gao 2019). Although, there is little correlation between the processes of immunity and senescence. However, because of the COVID-19 pandemic, the interdisciplinary connection between these fields is highly emphasized.

During exposure to stressful insults and certain physiological processes, cellular senescence occurs with a stable loss of growth potential and phenotypic alterations, especially the senescence-associated secretory phenotype (SASP) accompanied by a proinflammatory secretome (Calcinotto *et al.* 2019). In the early stages of senescence, chromatin and mtDNA fragments leaked to the cytosol to activate innate immune responses (Erdal *et al.* 2017; Fenech *et al.* 2011). After that, the IFN-I and inflammatory cytokines secreted by the host can engage in cell senescence in the late stage (Frisch and MacFawn 2020). In a word, innate immunity is affected by senescence and coordinates with it at the same time. Herein, we introduce the latest progress on innate immune pathways involved in cellular senescence. Subsequently, the association between immunity and senescence is illustrated. Finally, we discuss the potential therapeutic target based on innate immune responses for aging and age-associated diseases.

## CELL SENESCENCE

The senescence process is characterized by a generally irreversible cell-cycle arrest and other phenotypic alterations, like secretory features, macromolecular damage, and altered metabolism (Gorgoulis *et al.* 2019). Senescence exists at any life stage and is essential in embryogenesis, tissue homeostasis, and tumor suppression. Nevertheless, senescence may be the key ingredient of aging and age-associated diseases. Cellular senescence was first found in normal diploid cells undergoing an irreversible growth arrest after a fixed number of divisions by Hayflick and colleagues in 1961 (Hayflick and Moorhead 1961). After that, they noticed that this phenomenon was not observed in cells separated from malignant tumors, implying that senescence may be related to tumor suppression (Hayflick 1965).

Senescence is characterized by a permanent cell-cycle halt that results from exposure to damaging stimuli such as nutritional deprivation, genotoxic chemicals, hypoxia and dysfunction in the mitochondria, or abnormal proliferation such as oncogene activation. The p53 proteins and retinoblastoma (RB) family are important factors to enter senescent cell-cycle arrest in mammalian cells (Rodier and Campisi 2011). In senescent cells, ectopic expression of activated BRAF or RAS and persistent DNA damage upregulates the expression of CDK4/6 inhibitor p16INK4A and (CDKN2A) CDK2 inhibitor p21WAF1/Cip1 (CDKN1A) respectively (Sieben *et al.* 2018). Then RB family proteins activate persistently, repressing E2F transactivation and, ultimately, cell-cycle arrest, which can not be reversed by inhibiting p53 or RB family proteins (Beausejour *et al.* 2003; Sharpless and Sherr 2015). There are many reasons for this irreversibility, such as the influence of cytokines induced by senescent cells (Rodier and Campisi 2011), the heterochromatinization of E2F target genes (Salama *et al.* 2014), and the consistent generation of reactive oxygen species (ROS) (Takahashi *et al.* 2006). Besides, an additional protein, ARF, plays a crucial part in cell-cycle regulation, which is an alternate reading frame protein of p16 and can activate p53 as well (Sharpless and Sherr 2015).

The secretion of senescent cells is another prominent feature of senescence. Senescent cells typically produce numerous molecules, collectively called the senescent-associated secretory phenotype (SASP), including angiogenic factors, matrix metalloproteinases (MMPs), growth modulators, and various proinflammatory chemokines and cytokines (Coppe *et al.* 2010; Kuilman and Peeper 2009). Specifically, there are many signaling pathways participating in the production of SASP, mainly through enhancer remodeling and transcription factors activation, including mammalian target of rapamycin (mTOR), p38MAPK (Freund *et al.* 2011; Ito *et al.* 2017; Kuilman and Peeper 2009), NF-κB, GATA4, and C/EBPβ (Kang *et al.* 2015; Salama *et al.* 2014). Various signaling pathways contributing to SASP activation result from the different senescence inducers. Due to DNA damage, type I interferon response is triggered by cytoplasmic chromatin fragments (CCFs), and the inflammasome is activated by damage-associated molecular patterns (DAMPs) instead (Acosta *et al.* 2013; Davalos *et al.* 2013; Li and Chen 2018). Depending on the pathophysiological context, the senescence response can be useful or harmful. Early in our embryonic development, the SASP can help the morphogenesis of certain structures (Munoz-Espin *et al.* 2013) and initiate parturition (Menon *et al.* 2019). Besides, when tissue damage occurs, senescent cells can be found in the injured site transiently, where they function in wound healing and regeneration tissue repair, mainly by secretion of certain SASP factors (Ritschka *et al.* 2017; Sarig *et al.* 2019). One more critical function of SASP is in the process of immunity. Senescent cells can secrete SASP factors through autocrine and paracrine (Acosta *et al.* 2013; Coppe *et al.* 2010; Kuilman and Peeper 2009), which can activate immune responses, leading to the clearance of senescent cells (Krizhanovsky *et al.* 2008; Munoz-Espin and Serrano 2014). Considering the flip side, there are detrimental, pro-aging impacts of senescence because of the SASP. As reported, broad statistics from pharmacological interventions and transgenic rodent models consistently associate senescent cells with a high risk of various age-related pathologies, including tumorigenesis, cardiovascular diseases, and neurodegenerative disorders (Childs *et al.* 2017; Song *et al.* 2020). Chronic inflammation, mainly due to the SASP development, also termed "inflammaging" *in vivo*, is a main trigger for these illnesses (Franceschi and Campisi 2014). More and more senescent cells accumulate in various organs and tissues with age (Bussian *et al.* 2018; Childs *et al.* 2018), leading to compromised functional ability and more susceptibility to age-related disease.

### cGAS-STING

Genomic instability is a major contributor to aging. Persistent DNA damage is reported to lead to aging by causing senescence, cell death, and tissue dysfunction. DNA-sensor cyclic GMP-AMP synthase (cGAS) can bind extrachromosomal DNA fragments leaked into the cytoplasm in senescent cells (Schmitz *et al.* 2023), which leads to its liquid-liquid phase separation (Liu *et al.* 2023; Zheng and Gao 2023), conformational change, and activation. The activated cGAS utilizes GMP and AMP to produce the second messenger, cyclic GMP-AMP (cGAMP). cGAMP then binds a stimulator of interferon genes (STING) in the endoplasmic reticulum, activating STING and translocating to the Golgi apparatus (Motwani *et al.* 2019). Followed by the translocation of STING, IκB kinase (IKK) and TANK-binding kinase 1 (TBK1) are recruited and phosphorylate the NF-κB inhibitor IκBα and interferon regulatory factor 3 (IRF3). Then, NF-κB stimulates proinflammatory molecules due to phosphorylated IκBα and IFN-I and relevant downstream genes produced upon the transportation of active IRF3 to the nucleus (Corrales *et al.* 2017; Ishikawa *et al.* 2009). The following sections will explain the details about IFN I and NF-κB. Other sensors for cytosolic double-stranded DNA (dsDNA) include AIM2 and IFI16. They are members of the PYHIN family and can be induced by the interferon (IFN). AIM2 can assemble inflammasome to secret IL-1β and IL-18, whereas IFI16 induces IFN-β production upon binding dsDNA (Duan *et al.* 2011).

In cellular senescence, the abnormal accumulation of DNA in the cytoplasm is caused by several scenarios. One is cytosolic chromatin fragments (CCFs), due to the nuclear Lamin B1 loss in senescent cells, a main cause of genomic instability (Dou *et al.* 2015; Graziano *et al.* 2018). As reported, Lamin B1 over-expression can suppress the SASP (Rodier *et al.* 2009). In addition, long-interspersed element-1 (LINE-1 or L1), a major human retro-transposable element, is derepressed in senescent cells through downregulation in RB and an upregulation of the FOXA1 transcription factor, also serves as a source of cytosolic DNA (De Cecco *et al.* 2019). Its activation leads to cDNA accumulation, because of its high reverse transcriptase activity. Normally, LINE-1 elements are degraded by cytoplasmic DNases TREX1 and DNase2, decreased in senescent cells due to loss of E2F activity (Takahashi *et al.* 2018). A recent study shows that various retrotransposable elements, such as LINEs, ERV, IAP, and SINE B1, increase significantly in the aged mouse kidney and brain, where aging signals occur first (Ghanam *et al.* 2019). Furthermore, dysfunctional mitochondria can release mtDNA fragments, which leads to the activation of cGAS-STING signaling. Except for the nucleus, the only organelle having a genome is the mitochondria in the cell. There are many ways mtDNA can be leaked into the cytosol. In senescent cells, clearance of damaged mitochondria by mitophagy is not in time (Chen *et al.* 2020). Besides, the TFAM protein localized to the mitochondria can associate with mtDNA, forming nucleoid-like structures and imposing restrictions on mtDNA (Bonekamp and Larsson 2018; Ngo *et al.* 2014; Zierhut and Funabiki 2020). TFAM translocates to the cytosol during mitochondrial stress, helping cGAS bind mtDNA (West *et al.* 2015). The mtDNA in the cytosol increases by three to four fold upon TFAM depletion. Moreover, mtDNA can enter the cytoplasm through pore formation by BCL-2 associated X protein (BAX) (McArthur *et al.* 2018) and voltage-dependent anion channel (VDAC) (Kim *et al.* 2019a). The accumulation of extranuclear DNA species, including CCFs, cDNA, and mtDNA in senescent cells, elicit cGAS-STING signaling to promote full-spectrum SASP expression. CCFs, cDNA, and mtDNA constitute the accumulation of extranuclear DNA species in senescent cells, by which cGAS-STING signaling initiates.

Emerging evidence suggests a connection between the cGAS-STING pathway and premature aging diseases. One of them is Hutchinson Guilford Progeria (HGPS), and the cause of this disease is truncation of Lamin A (progerin). Replicative stress induced by progerin can lead to genomic instability, further activating the cGAS-STING pathway to initiate IFN responses (Kreienkamp *et al.* 2018). Meanwhile, the cGAS-STING pathway participates in neurodegenerative disorders, such as Ataxia-Telangiectasia (A-T), Huntington’s disease (HD), and Parkinson’s disease (PD) (Paul *et al.* 2021). It is especially noteworthy that the YAP/TAZ-cGAS-STING signaling conduit finds a link between mechanosensing and innate immunity. YAP/TAZ activity and cellular mechano-signaling are decreased upon physiological aging, leading to unscheduled cGAS-STING activation (Sladitschek-Martens *et al.* 2022). Significantly, several effective drugs, such as aspirin and quinacrine, reduce the cGAS–STING signaling transduction. Mechanically, aspirin can directly bind and acetylate cGAS to keep cGAS inactive (Dai *et al.* 2019), while quinacrine disrupts the conformation of dsDNA to influence the cGAS activity indirectly (Lama *et al.* 2019). Research on new inhibitors is being actively carried out.

### TOLL-LIKE RECEPTORS (TLRs)

Toll-like receptors (TLRs) are the first described PRRs in the innate immune system, which are crucial in inflammatory responses (Fitzgerald and Kagan 2020; Iwasaki and Medzhitov 2010). They were first identified in Drosophila (Schneider *et al.* 1994) and demonstrated to play important roles in innate immunity by later studies (Gay and Keith 1991). Among TLRs, TLR4 was first identified. After the discovery of TLR4, many PRRs and corresponding ligands were disclosed (Wright 1999). There are 13 different TLRs (TLRs 1–13) discovered in mammals until now (Kawai and Akira 2010), and 10 of these (TLRs 1–10) function in humans. Upon stimuli, TLRs recognize specific DAMPs and PAMPs to initiate signaling transduction (Anthoney *et al.* 2018; Satoh and Akira 2016). According to different adaptor proteins, there are two TLR signaling pathways, namely MyD88-dependent and MyD88-independent (Kawai and Akira 2007). Most TLRs depend on the MyD88-dependent pathway (von Bernuth *et al.* 2008). In this pathway, secretion of various pro-inflammatory factors, such as IL-6, IL-1, and TNF-α, are induced by MyD88 signaling, leading to inflammation (Gay *et al.* 2014; Kawai and Akira 2011). First, TLRs interact with the C-terminus of MyD88 through the intracellular Toll/interleukin-1 receptor (TIR) domain, and IL-1R-related kinase 4 (IRAK4) is recruited to the N terminus of MyD88 (De Nardo *et al.* 2018). Then, the central kinase domain of MyD88 undergoes autophosphorylation, which leads to the activation of IRAK1 and IRAK2. After that, transforming growth factor (TGF)-β-activated kinase 1 (TAK1) and two TAK-binding proteins (TAB1 and TAB4) form a complex with ubiquitin ligase TNF receptor associated factor 6 (TRAF6), by which IκB kinase (IKK) complex is activated through phosphorylation. Phosphorylation of IκB leads to its degradation through the ubiquitin-proteasome pathway (Hacker *et al.* 2006; Verstak *et al.* 2009), which releases the transcription factor NF-κB. The unleashing of NF-κB causes it to translocate from the cytoplasm to the nucleus and initiates the expression of inflammatory cytokines (Cohen and Strickson 2017; Kawai *et al.* 2004). Furthermore, TLR3 and TLR4 mediate the MyD88-independent pathway. TLR4 recruits and activates TIR-domain-containing adaptor inducing interferon-γ (TRIF) and TRIF-related adaptor molecule (TRAM), leading to the activation of IRF3 and NF-kB, eventually stimulating IFN-I production. TLR3 activation also needs adaptor TRIF, but the downstream signal molecules recruited are TBK1 and IKKε (Arancibia *et al.* 2007; Okun *et al.* 2009).

Numerous studies claim that TLRs are involved in aging and aging-related disorders, such as neurodegenerative diseases, cardiovascular diseases, and disc degeneration. The progress of neurodegeneration is caused by neuroinflammation. In this respect, TLRs are important in CNS disorders, especially TLR4 (Azam *et al.* 2019). The TLR4/NF-kB-signaling pathway can lead to many kinds of inflammatory cytokines, such as MMP-9 and COX-2, which may be a cause of secondary brain injury (Hua *et al.* 2007; Kerfoot *et al.* 2004; Lucas *et al.* 2006; Wang *et al.* 2000). In the clinic, loading nanoparticles with quercetin can prevent AD progression as a result of suppressing TLR4-involved pathway (Testa *et al.* 2014). Meanwhile, quercetin nanoparticles also downregulate inflammatory cytokine secretion by inhibiting the expression of TLR2 and TLR4 (Bhaskar *et al.* 2011). Based on these observations, TLR4 may be a potential target to develop novel strategies for neurodegenerative disorders. Many studies have shown that different TLRs are involved in the process of atherosclerosis through distinct mechanisms (Li *et al.* 2020; Wolf and Ley 2019). TLR7 can inhibit inflammation in atherosclerosis by secreting TNF-α and IL-10, so it is regarded as a prognosis marker in severe atherosclerosis patients (Karadimou *et al.* 2017). Besides, cell-free DNA (cf DNA) recognized by TLR9 can lead to the development of vascular inflammation, which induces the formation of initial atherosclerotic plaques (Fukuda *et al.* 2019). TLR5 upregulates the expression of proinflammatory factors by inducing the activation of NADPH oxidase 4 (Nox4), which is crucial for atherosclerotic intimal hyperplasia (Kim *et al.* 2016; Kim *et al.* 2019b). TLRs are also closely associated with disc degeneration and virus-induced aging. It is reported that TLR2 can induce senescence in intervertebral disc cells, and o-vanillin may be a suitable drug for these patients (Mannarino *et al.* 2021). Similarly, another study gives the novel hyaluronic acid granular hydrogel as a new strategy for osteoarthritis progression via suppressing TLR2-mediated cellular senescence (Zhang *et al.* 2023). Furthermore, TLR3 causes senescence in patients infected with COVID-19 and tremendously increases SASP production (Tripathi *et al.* 2021).

## IFN-I

Interferons were first discovered in 1957 by Isaacs and Lindenmann (Isaacs and Lindenmann 1957). They are crucial molecules playing important roles in both innate and adaptive immunity. Through various pattern recognition receptors (PRRs), the process of innate immunity initiates, and the type I IFN genes are induced rapidly. These receptors recognize molecules from pathogens known as pathogen-associated molecular patterns (PAMPs), such as cytosolic DNA, double-stranded RNA (dsRNA), flagellin, bacterial lipoproteins, and bacterial lipopolysaccharides (Amarante-Mendes *et al.* 2018). In addition to PAMPs, damage-associated molecular patterns (DAMPs) presented by damaged cells can also be perceived by certain PRRs (Roh and Sohn 2018). Many other non-classical IFN-I stimuli were found recently, such as DNA damage, FAS/FASL activation (Qadir *et al.* 2020), and endogenous cytosolic chromatin fragments. These non-classical IFN-I stimuli link innate immunity and senescence together (Deng *et al.* 2014; Sistigu *et al.* 2014; Yu *et al.* 2015). Herein, we briefly review the signaling mechanisms of IFN-I induction induced by PRRs. First, endosomal Toll-like receptor (TLR3), as well as cytosolic receptors retinoic acid-inducible gene-I (RIG-I) and melanoma differentiation-associated protein 5 (MDA5), recognize dsRNA. Cytosolic TLR9 and cGAS recognize DNA. Primarily, pattern recognition receptors converge signals upon IKK-family kinases, leading to the phosphorylation and activation of two downstream transcription factors, IRF3 and NF-κB. Upon activation, these factors directly induce the IFN-β gene and various antiviral target genes expression, including ISG15 (Villarroya-Beltri *et al.* 2017) and many antiviral genes containing MxA/B, IFIT- and IFITM-family genes (Ashley *et al.* 2019). After that, secreted IFN-β from upstream pathways activates another receptor called the IFNAR1/2 receptor. Then, Janus kinase phosphorylates STAT1 and STAT2, resulting in a canonical complex of STAT1/STAT2/IRF9 (ISGF3) formation. Certain genes, called interferon-stimulated genes (ISGs) expressing, contain ISGF3 response elements (ISRE), leading to a second wave of IFN-β production (Ng *et al.* 2016). Meanwhile, autocrine IFN-I causes cell-cycle arrest through p53 and Rb checkpoint pathways (Braumuller *et al.* 2013; Sangfelt *et al.* 1999). In addition, two ISGs can contribute to the cycle. While IFI16 can repress c-myc, IFI202a/b gene products can interfere with the transactivation of MYC and E2F proteins (Song *et al.* 2010). Besides, in many cases, apoptosis can serve as an efficient innate immune mechanism resulting from poly-ubiquitinated IRF3, named RLR-induced IRF-3-mediated apoptosis (RIPA) (Chattopadhyay *et al.* 2016).

There has been plenty of evidence pointing out that IFN-I plays a strong part in DNA damage signaling and senescence (Frisch and MacFawn 2020). The up-regulation of ISGs was first found in senescent fibroblasts in 1995, which can be inhibited by IFN-β-neutralizing antibodies (Tahara *et al.* 1995). Then, in 2003, through oligonucleotide microarrays, Michael Tainsky's laboratory found that among the genes upregulated in senescent cells and down-regulated in immortalized cells simultaneously, 46% were IFNs or ISGs (Kulaeva *et al.* 2003). In the same year, Tadatsugu Taniguchi's laboratory found that in response to DNA damage, IFN-I increased the expression of p53 and cooperated with stressful events to activate p53 (Takaoka *et al.* 2003). IFN-I can engage in p53 signals, and the important role p53 plays is initiating senescence from growth arrest, but p53 is not necessary for the secretion of SASP (Coppe *et al.* 2008), which is exactly the stage where IFN-I comes into play. Later reports showed that cell senescence could be induced by prolonged IFN-β treatment in fibroblasts, resulting in various DNA damage response (DDR) features containing phosphorylation of γ-H2AX foci (indicative of unrepaired double-strand DNA breaks), ATM, p53, CHK2, and increased ROS (Moiseeva *et al.* 2006). The senescence was dependent on ATM and ROS. Meanwhile, the laboratory of Serge Fuch (Yu *et al.* 2015) found that DSB induced by FokI nuclease fusion proteins resulted in IFN-β production and the induction of phospho-STAT1. Furthermore, this phenomenon depended on ATM, IRF3, and IKKα/β activation. More surprisingly, IRF3 was found to resident in chromatin repair foci, which provided a molecular basis.

A clinical study observed that there was an increase in DNA repair foci and high expression of IFN-β in fibroblasts isolated from Terc-deficient mice or progeria patients (Yu *et al.* 2015). Prominently, neutralizing antibodies targeting IFN-β can partially combat the effects of senescence, whether in the normal senescent and progeria fibroblasts. This laboratory also demonstrated that knockout of the IFNAR1 receptor partially survived the phenotypes resulting from the shorter telomeres in stem cells, with distorted intestinal crypt-and-villus structure. These results suggested that IFN-I signaling participated in stem cell senescence. In agreement with previous studies, IFN-I also upregulates DDR because knockout of IFNAR1 leads to decreased p53 activation and the senescence markers, p21WAF/CIP, and p16INK4a. Research suggests that Terc^-/-^ mice are more susceptible to suffering a premature aging phenotype after incubating for some generations. Prominently, the IFNAR1 knockout relieved this phenotype and increased life span significantly (Yu *et al.* 2015). In conclusion, not only IFN-I contributes to DDR-induced senescence, but also DDR contributes to IFN-I. Besides, as membrane-bound IFNAR1 gradually decreases and IFN-I signaling is inhibited with age, melanoma progression commonly occurs (Araya and Goldszmid 2017; Fuchs 2013). It is conceivable that IFN-I and IFNAR1 are likely potential targets for increasing the health span of older adults.

### NF-κB

Inflammaging is defined as a chronic, sterile, low-grade inflammation (Franceschi *et al.* 2000), which does not simply increase with age but has a highly positive correlation with a decline in health, age-related diseases, and mortality risk (Arai *et al.* 2015; Bruunsgaard 2006; Krabbe *et al.* 2004). Inflammaging results from a chronic gathering of senescent cells with age, which produce large amounts of SASP, including chemokines, proinflammatory cytokines, metalloproteases, soluble receptors, growth factors, and certain protease inhibitors (Coppe *et al.* 2010; Freund *et al.* 2010; Malaquin *et al.* 2016). The secretion of SASP varies in certain signaling pathways from cell to cell, but the central one is the same: the transcription factor NF-κB (Chien *et al.* 2011; Meyer *et al.* 2017; Salminen *et al.* 2012). It plays an important role in regulating inflammation response in the immunity process, and a later study discovered it to be crucial in SASP generation.

There are a wide variety of inducers leading to the activation of NF-κB, containing ionizing radiation, bacterial lipopolysaccharides, reactive oxygen species (ROS), abnormal DNA and RNA, and cytokines like interleukin 1-beta (IL-1β), and tumor necrosis factor-alpha (TNF-α) (Zhang *et al.* 2017). During the resting state, NF-κB localizes in the cytoplasm, forming a complex with inhibitors of NF-κB (IκBs), which maintains the inactive stage of NF-κB. In response to stimulation, IκB kinase (IKK) phosphorylates IκBs for degradation, resulting in the transport of NF-κB to the nucleus. After that, larger quantities of gene transcription are initiated, which contributes to cell proliferation, inflammation, and apoptosis (Oeckinghaus and Ghosh 2009), which is termed the "canonical pathway" of NF-κB because NF-κB essential modulator (NEMO) participates in the process, which is a part of the IKK complex (Hariharan *et al.* 2021). Except for cGAS-STING signals, the upstream canonical pathway includes dsRNA-dependent protein kinase (PKR) (Williams 1999) and myeloid differentiation primary response 88 (MyD88) (D'Acquisto *et al.* 2002). The PKR can also participate in the "noncanonical pathway" of NF-κB activation by TNF-α. This pathway can lead to a chronic and persistent inflammation response, contrasting with the canonical one (Dorrington and Fraser 2019). Besides, the serine-threonine protein kinases MAPKs activate NF-κB in a noncanonical way. During pathophysiological and stress, MAPKs activated by P38 mitogen mediate the production of IL-1β and TNF-α (Battagello *et al.* 2020). To achieve full activation, The Janus Kinase (JAK)-transcription factor (STAT) pathway needs to be activated by IL-6, a remarkable marker in senescent cells. Upon IL-6 sensed, STAT3 is phosphorylated and then translocated to the nucleus to decrease the production of IFN-γ and release inflammatory factors (Brasier 2010). Apart from the signaling above, cGAS-STING can also control innate immunity by transcribing acute-phase serum amyloids (A-SAAs) and Toll-like receptor 2 (TLR2) mediated by NF-κB in senescent cells. In this pathway, TLR2 recognizes A-SAAs, leading to the further activation of NF-κB with the secretion of proinflammatory SASP molecules, which serves as a positive feedback loop in senescence. As reported, overexpression of TLR2 causes cell cycle arrest, leading to SA-β-gal production, while TLR2 deficiency decreases the expression of p16, p21, and SASP (Hari *et al.* 2019).

Recently, studies have shown that NF-κB-mediated inflammation directly affects DNA damage response (Fang *et al.* 2014; Hinz *et al.* 2010; Kang *et al.* 2015). When DNA damage occurs, ATM and RAD3-related (ATR) and ataxia-telangiectasia mutated (ATM) are recruited to the DNA lesion by single-stranded DNA breaks and DSBs, respectively (d'Adda di Fagagna 2008). During the resting state, the p62 protein can target transcription factor GATA4 for degradation by autophagy. Upon stimuli, p62 is restrained by ATM or ATR, providing the basis for GATA4 to induce inflammation mediated by NF-κB. In addition, via translocation to cytoplasm, ATM can activate TRAF6 to initiate NF-κB signals further. Prominently, ATM can activate STING without cGAS, contributing to NF-κB activation (Dunphy *et al.* 2018). Additionally, activated ATM can degrade IκBα, further leading to the phosphorylation of RELA (p65), resulting in NF-κB activation. ATM has a dramatic effect on the process. To sum up, DNA damage has increasingly become a crucial regulator of inflammation, binding inflammation and aging together (Zhao *et al.* 2023).

The study on NF-κB pathways may provide new drug targets for aging and aging-related illnesses. For this problem, a well-studied antidiabetic drug, metformin, is wildly used to relieve inflammaging. Metformin can restrain NF-κB signaling and the production of proinflammatory factors downstream (Moiseeva *et al.* 2013). Meanwhile, it can activate autophagy to clear up senescent cells (Bharath *et al.* 2020). Recent studies showed that metformin can promote autophagy, improve mitochondria function in CD4^ + ^ T cells isolated from old donors in vitro, and restrict the age-related proinflammatory profile of Th17. Furthermore, many reports focus on the roles of metformin in reducing autoimmunity and supporting healthy immune function. Besides, there are new therapies targeting IL-6 to suppress NF-κB pathways. Dexamethasone, a steroid hormone, can retain NF-κB in the cytosol by increasing IκB expression (Auphan *et al.* 1995) and inhibit the activity of NF-κB by interfering with IL-6 secretion (Buhl *et al.* 2019; Ge *et al.* 2018; Laberge *et al.* 2012). Tocilizumab and sarilumab, two antibodies targeting the IL-6 receptor, have been utilized in clinics as an ideal therapeutic method for severe patients (Wise 2020).

## NLRP3

Acting as a signal complex, it is obvious that the inflammasomes play an important role in various inflammation-associated diseases. There are various inflammasomes, such as NLRP1, NLRP3, NLRC4, IPAF, and AIM2. Among them, the NLRP3 inflammasome garners particular attention for its capacity to identify numerous DAMPs and PAMPs and to resist the invasion of exotic microbes. The NLRP3 inflammasome contains three members: nucleotide-binding domain leucine-rich repeat (NLR) and pyrin domain containing receptor 3 (NLRP3), apoptosis-associated speck-like protein containing a caspase recruitment domain (ASC), and pro-caspase-1 (Yang *et al.* 2019). NLRP3 monomers gather in oligomers, then recruit and bind with ASC upon danger stimuli. Afterward, pro-caspase-1 joins the signal complex via ASC (Yang *et al.* 2019; Zahid *et al.* 2019). The activation of NLRP3 inflammasome requires PAMPs and DAMPs. Derived from immune cells stimulated by damaged tissues, they are termed DAMPs, whereas sugar conjugates and glycans compose PAMPs. There have been many studies on PAMPs and DAMPs with the NLRP3 inflammasome recently. For instance, NLRP3 inflammasome can be activated by the cumulation of uric acid (UA) (Martinon *et al.* 2006) and the expression of cathepsin B (CTSB) (Tang *et al.* 2018). Furthermore, sensed by TLR4 localized on the cellular plasma membrane, lipopolysaccharides (LPS) from Gram-negative bacteria activate NF-κB to promote the activation of the NLRP3 inflammasome, sending the signals to caspase-1 (Yu *et al.* 2017). After that, caspase-1 cleaves pro-IL-18 and pro-IL-1β to generate IL-18 and IL-1β, and then the inflammatory response initiates to secrete plenty of proinflammatory factors (Yang *et al.* 2019).

Mounting evidence shows that NLRP3 participates in the process of many age-related symptoms. Regarding aging-related fibrosis, the alveolar macrophages isolated from old mice with bleomycin (BLM)-induced lung injury have a higher activation of NLRP3 inflammasome and secret more IL-18 and IL-1β, compared to young mice (Stout-Delgado *et al.* 2016). Notably, chronic aging-related diseases, like nonalcoholic fatty liver disease, lead to metabolic disorders, causing hyperactivation of NLRP3 inflammasomes and severe liver fibrosis (Liu *et al.* 2022). Consequently, the NLRP3 inflammasome is one of the main causes of fibrosis development. Not only that, it also contributes to organ aging. In the ovary, the expression of NLRP3 increases with age in WT mice, which is not observed in the littermate control of NLRP3 KO mice. Knockout of NLRP3 can ameliorate the function of the ovary and increase pregnancy rates obviously (Navarro-Pando *et al.* 2021).

Controlling the activation of inflammatory response is crucial for delaying the natural aging process and staying healthy. NLRP3 may be an appropriate target to be inhibited, by which inflammation is suppressed (Zahid *et al.* 2019). Recently, in macrophages from old mice, deacetylation of NLRP3 via SIRT2 suppressed age-related inflammation resulting from NLRP3 activation (He *et al.* 2020). The expression of SIRT2 reduced with age, and the NLRP3 inflammasome was activated by mitochondrial dysfunction, destroying the regenerative ability of HSCs (Luo *et al.* 2019). In addition, MCC950, an inhibitor of NLRP3, can inhibit the mammalian targets of rapamycin (mTOR) pathway in aging mice, thereby increasing autophagy and reducing the inflammation response, finally improving liver dysfunction (Marin-Aguilar *et al.* 2020). More importantly, MCC950 can reduce liver, lung, and kidney fibrosis in aging mice. Besides, MCC950 can increase the fertilization ratio in female mice to the same level in Nlrp3^-/-^ mice (Navarro-Pando *et al.* 2021). MCC950 has significant efficacy and specific features in age-related inflammation in mice, but it still has a long way to go for human clinical trials.

## SUMMARY AND PERSPECTIVES

From the modern point of view, aging means symptoms of degenerative changes, leading to many chronic diseases, including neurological degeneration, fibrosis, cardiovascular disease, diabetes, and cancer (Cai *et al.* 2022). In contrast, cellular senescence serves as the main cause of aging. Upon stimuli, such as exposure to genotoxic agents and oncogene activation, cells enter a stage with irreversible growth arrest, accompanied by secretory features, oxidative damage of biomacromolecules, and metabolic changes. The SASP secreted by senescent cells recruits immune cells to recognize and clear up damaged cells, finally maintaining the balance of the immune system (Kohli *et al.* 2021). However, immune system dysfunction causes senescent cell accumulation, persistent tissue damage, and, more severely, chronic low-grade inflammation, termed inflammaging (Teissier *et al.* 2022). The key strategy to suppress senescence is improving the immune function and reducing the inflammation response. In this review, we introduce the signaling pathways involved in senescence. First, we briefly give the basic conception of senescence, including the initiation of senescence, the characteristics of different stages, and the classic pathways contributing to senescence. Second, we summarize the key innate immune pathways participating in senescence, including cGAS-STING pathways, TLRs pathways, IFN-I and downstream pathways, NF-κB pathways, and NLRP3 inflammasome pathways. In senescent cells, the occurrence and regulation of these pathways, different from normal responses, are elaborated in the following paragraphs. Each part is not independent and is linked to all the others. cGAS-STING pathways play important parts in connecting cellular damage and inflammation, the activation of which can lead to both IFN-I and inflammatory factors. Similarly, TLRs-mediated signal transduction pathways can both activate proinflammatory cytokine responses and upregulate IFN-I and ISGs expression, integrating innate immune responses. While IFN-I and ISGs can amplify the damage signals and trigger the clearance of senescent cells. Meanwhile, it can work in concert with NF-κB pathways also. Inflammatory factors secreted upon NF-κB activation serve as triggers for the downstream of IFN-I, like IL-6. Besides, NF-κB pathways can provide the first signal of the NLRP3 activation. At the end of each section, we discuss the potential strategies targeting the key molecules, including cGAS, STING, TLRs, IFN-I, IL-6, and NLRP3. Some of the drugs above have strong and stable effects in animal tests, but more research is needed to conduct human trials.

**Figure 1:**
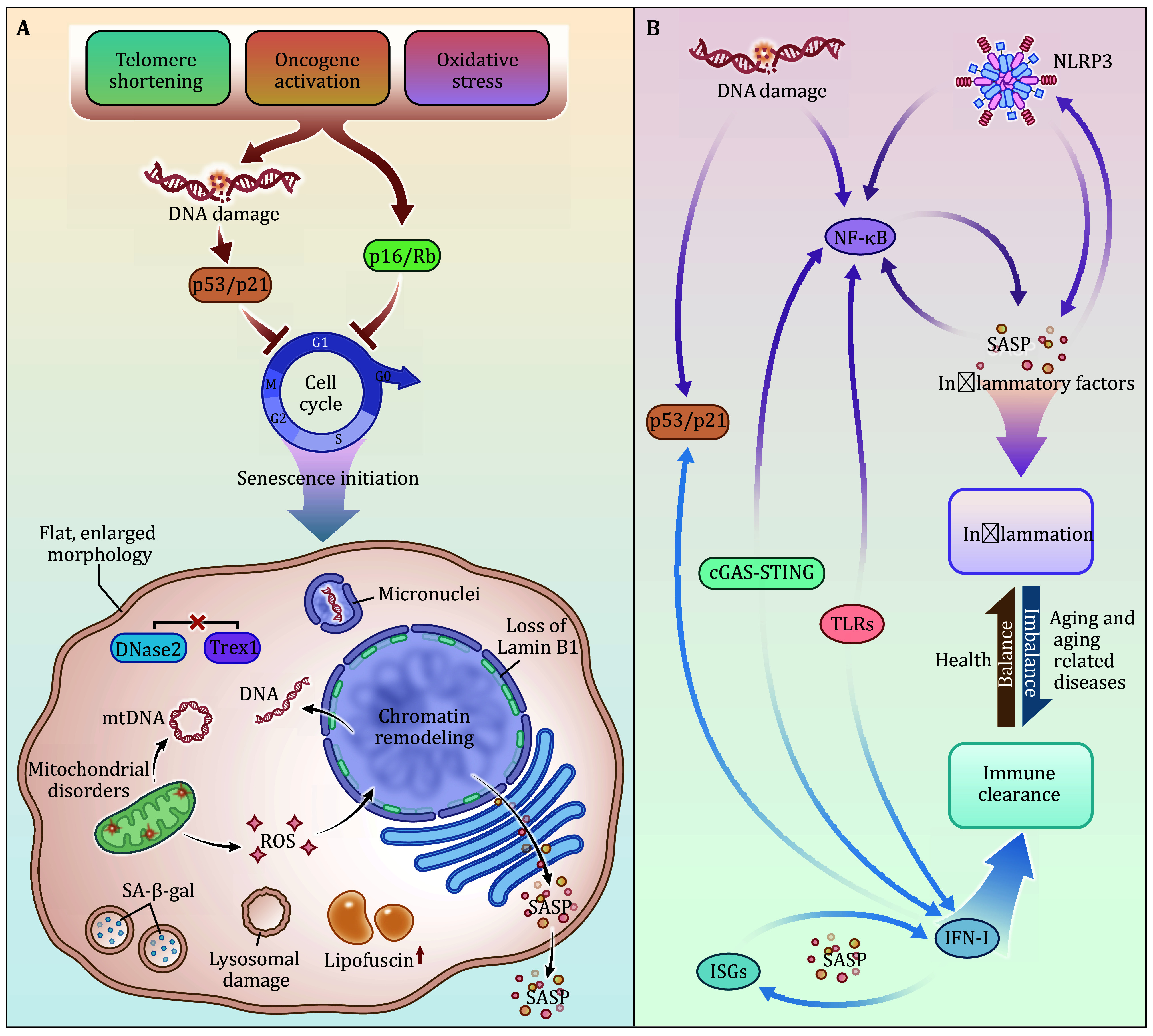
The interaction between senescence and innate immune signaling pathways. **A** The mechanisms of cell senescence. During senescence, p53-p21 and p16-Rb pathways are firstly activated by numerous stressors, leading to irreversible cell-cycle arrest. Then, a series of changes, such as cytoplasmic DNA accumulation, mitochondrial disorders, and lysosomal damage, occur. Additionally, SA-β-gal activity, lipofuscin accumulation, and SASP production are induced and regarded as senescent markers. **B** cGAS-STING, TLRs, and NLRP3 inflammasome participate in the process of senescence and promote the SASP through the production of inflammatory factors, IFN-I, and downstream ISGs, respectively. In addition, NF-κB can be stimulated by DNA damage, and IFN-I can regulate the P53 signaling pathway, which is directly involved in the signaling pathway of senescence. The balance of inflammation and immunity is essential for the body to achieve healthy aging

In this review, we summarize the crucial innate immune signaling pathways associated with cellular senescence and elaborate on the relationship between cellular senescence and innate immunity (Fig. 1). Senescence, IFN-I, and inflammation all have one thing in common: they are all double-edged swords with both advantages and disadvantages. On one hand, aging removes damaged cells, facilitates tissue recovery, and inhibits carcinogenesis. On the other hand, it can also cause premature aging and a series of aging-related diseases. IFN-I responses, on the one hand, can help the body resist pathogen invasion and help the immune clearance of target cells. On the other hand, it can also cause autoimmune diseases. Likewise, inflammation can secrete inflammatory factors to help the host kill pathogenic microorganisms, leading to apoptosis and cell death but disrupting immune homeostasis. At present, the study of cellular senescence is in the ascendant, and the relationship with immunity has gradually received attention because of the COVID-19 pandemic. According to current research, immunity is involved in cellular senescence, mainly in the later stage, playing a regulatory role (Frisch and MacFawn 2020). Whether IFN-I and inflammatory signaling pathways are involved in the early stages of aging warrants further study. Many studies have only found the link between immune molecules and senescence, but the mechanism has yet to be addressed. For example, cGAS-STING is involved in the process of aging regulated by YAP/TAZ, but the specific mechanism has not been discovered (Sladitschek-Martens *et al.* 2022). In addition, it is not known whether the senescence pathway is directly involved in immune regulation and whether p21, p16, and DNA damage-related molecules are directly involved. Overall, the crosstalk between senescence and innate immune signaling pathways remained to be explored.

## Conflict of interest

Jinxiu Hou, Yi Zheng and Chengjiang Gao declare that they have no conflict of interest.
